# Factors associated with compliance among users of solar water disinfection in rural Bolivia

**DOI:** 10.1186/1471-2458-11-210

**Published:** 2011-04-04

**Authors:** Andri Christen, Gonzalo Duran Pacheco, Jan Hattendorf, Benjamin F Arnold, Myriam Cevallos, Stefan Indergand, John M Colford, Daniel Mäusezahl

**Affiliations:** 1 Department of Public Health and Epidemiology, Swiss Tropical and Public Health Institute, P.O. Box, 4002 Basel, Switzerland; 2 University of Basel, Basel, Switzerland; 3 Division of Epidemiology, School of Public Health, University of California, Berkeley, CA 94720-7360, USA

## Abstract

**Background:**

Diarrhoea is the second leading cause of childhood mortality, with an estimated 1.3 million deaths per year. Promotion of Solar Water Disinfection (SODIS) has been suggested as a strategy for reducing the global burden of diarrhoea by improving the microbiological quality of drinking water. Despite increasing support for the large-scale dissemination of SODIS, there are few reports describing the effectiveness of its implementation. It is, therefore, important to identify and understand the mechanisms that lead to adoption and regular use of SODIS.

**Methods:**

We investigated the behaviours associated with SODIS adoption among households assigned to receive SODIS promotion during a cluster-randomized trial in rural Bolivia. Distinct groups of SODIS-users were identified on the basis of six compliance indicators using principal components and cluster analysis. The probability of adopting SODIS as a function of campaign exposure and household characteristics was evaluated using ordinal logistic regression models.

**Results:**

Standardised, community-level SODIS-implementation in a rural Bolivian setting was associated with a median SODIS use of 32% (IQR: 17-50). Households that were more likely to use SODIS were those that participated more frequently in SODIS promotional events (OR = 1.07, 95%CI: 1.01-1.13), included women (OR = 1.18, 95%CI: 1.07-1.30), owned latrines (OR = 3.38, 95%CI: 1.07-10.70), and had severely wasted children living in the home (OR = 2.17, 95%CI: 1.34-3.49).

**Conclusions:**

Most of the observed household characteristics showed limited potential to predict compliance with a comprehensive, year-long SODIS-promotion campaign; this finding reflects the complexity of behaviour change in the context of household water treatment. However, our findings also suggest that the motivation to adopt new water treatment habits and to acquire new knowledge about drinking water treatment is associated with prior engagements in sanitary hygiene and with the experience of contemporary family health concerns.

Household-level factors like the ownership of a latrine, a large proportion of females and the presence of a malnourished child living in a home are easily assessable indicators that SODIS-programme managers could use to identify early adopters in SODIS promotion campaigns.

**Trial Registration:**

ClinicalTrials.gov: NCT00731497

## Background

Systematic reviews of water, sanitation, and hygiene interventions in developing countries suggest that improved drinking water or hand hygiene interventions could prevent between 20% and 35% of the global 3.5 billion diarrhoea episodes per year [1-5]. The evidence to date led the World Health Organisation (WHO) to conclude that household water treatment (HWT) is the most cost-effective approach to reach the United Nations millennium development target 7c of halving the number of persons with no access to safe water (WHO report 2002).

However, the majority of evidence has been collected in controlled intervention trials that document efficacy of HWT by improving water quality and reducing diarrhoeal disease in developing countries [6]. These tightly controlled experiments typically last fewer than six months and include both subsidized (or free) materials and high levels of behaviour reinforcement [7]. Evidence for effectiveness on a larger scale and sustained use are rarely addressed by HWT studies [4,8], but such evidence is necessary to guide global efforts to scale up HWT [9,10].

Solar water disinfection (SODIS) is one of the simplest and cheapest technologies for household water disinfection. The method relies on disposable translucent plastic bottles of 1-2 litres in which pathogen-containing water is purified by the combined pathogen-inactivating effects of solar radiation and heating [11,12]. Laboratory experiments proved its efficacy in improving the quality of water [12-14]. The method is widely disseminated in developing countries to improve health in settings where safe drinking water is not available. Despite this widespread promotion, only a few field studies assessed its health impact and evidence on acceptance, regular use, and scalability of the method is scarce and inconclusive [9,10,15-18]. Recent studies demonstrate that SODIS promotion is unlikely to reduce diarrhoea in children below 5 years of age if there are low adoption rates and limited long-term use by the target population [6,15,19,20]. It is therefore, important to identify and understand the mechanisms that attenuate the health impacts of SODIS despite its high efficacy for improving water quality under ideal conditions [12,21].

One challenge of assessing the effectiveness of SODIS implementation is the lack of a reliable, unbiased and accepted indicator to measure SODIS-use. Compliance with the SODIS-intervention (e.g. consumption of the SODIS-treated water) is an important indicator of success of the implementation strategy. To our knowledge, none of the SODIS studies that measured its effectiveness to improve water quality for preventing diarrhoea assessed determinants of compliance directly. To date, the most common end-points used to assess SODIS-use rely on self-reported use or the direct observation of water-filled plastic bottles exposed to sunlight [16,18,22-25]. Indicators are often assessed once, usually at the end of the intervention, and the reliability of these indicators is unknown. Self-reported use in the context of an interview is known to produce inflated results due to reporting bias [26-29]. Togouet et al. use five measures of self-reported use, direct observation and interviewer opinion to create a 0-5 score to classify 'non-users,' 'irregular users,' and 'regular users' [18]. However, this approach to user classification uses a score that weights all components equally, and forces the investigator to subjectively choose cut points in that score. There is a need for objective methods to classify households into distinct SODIS user groups.

In this article we present a detailed analysis of SODIS compliance among recipients of a SODIS-intervention who participated in a community-randomised, controlled SODIS trial (cRCT) in rural Bolivia (BoliviaWET). The trial detected no statistically significant reduction in diarrhoea in children under age 5 with an overall SODIS compliance of 32% based on community-health worker assessment [15], a measure that was more conservative than indicators applied in studies with high SODIS-usage rates [16-18]. Here, we use weekly data collected over 12 months from the SODIS compliance monitoring and the SODIS promotion campaign of BoliviaWET to objectively classify households into distinct SODIS-use groups using principal components and cluster analysis. We then use the classified groups to describe the household determinants and campaign implementation factors that are associated with the adoption and utilisation of SODIS in our setting.

## Methods

Twenty-two communities from the Totora district, Cochabamba department, Bolivia were included in the cRCT and randomised to receive the SODIS as a HWT. Data of 216 of 225 households enrolled in the 11 intervention communities of the cRCT were included in this analysis. We excluded 9 households from the analysis that were monitored for fewer than 6 weeks over the 12 month follow-up period.

### Study site

The Totora district covers an area of 2000 km^2^. Community settlements are widely dispersed at altitudes between 1700 and 3400 m. The majority of the ethnically homogeneous Quechua population are subsistence farmers that grow potatoes, wheat and maize. Households keep livestock for their own consumption and for sale. Families typically live in small compounds of three buildings with mud floors, with several persons sleeping in the same room. Only 18% of the homes have a latrine. Most residents defecate in the nearby environment. Unprotected springs are the predominant drinking water sources.

### SODIS campaign

The campaign had two main objectives: i) to create demand for safe drinking water, and ii) to establish a sustainable application of SODIS as a drinking water disinfection method at household level. A non-governmental organisation, Project Concern International (PCI), implemented the campaign. PCI was well known in the study communities from prior work, and at the time of the intervention had experience promoting SODIS in rural Bolivia. PCI introduced SODIS during an intensive 15-month period that started 3 months before the 12-month epidemiologic field trial and continued for three months after the trial in the communities of the control arm.

The implementation in intervention communities was standardised at the community and household levels (see Additional file 1). The campaign introduced SODIS along with water and sanitation hygiene messages to study communities through participative interactions during district events, community events and personal home visits. District-level stakeholders (farmers' union, local government officials, health and school system representatives) as well as formal and informal community leaders were involved in promoting SODIS. In the field, PCI staff and local community advocates (health personnel and teachers) promoted SODIS through focus groups, community- and school events, community training workshops and monthly home visits. Community events were held at least monthly. All community members were invited to these events where they were trained and motivated to practice SODIS daily in their homes.

Experienced health promoters from PCI conducted motivational home visits to empower participants to disinfect their drinking water before consumption and to adopt or improve hygiene habits to create a less contaminated home environment. The motivational home visit strategy was based on participatory hygiene and sanitation transformation methodologies and motivational interviewing [30-32].

### SODIS-use assessment

Data regarding SODIS-use were collected by community-based field workers who were integrated into the community and were not involved in any SODIS promotion or implementation activities. The field staff was extensively trained in interviewing and epidemiological observation techniques, data recording, and participatory community motivation approaches. Field staff recorded SODIS-use indicators during weekly home visits with a structured, inconspicuous, observational protocol. In addition, field staff recorded self-reported SODIS-use three months after the beginning and at the end of the intervention campaign (after 15 months).

PCI measured study participants' degree of exposure to the SODIS implementation campaign by registering the individual attendance during SODIS promotional events.

In order to arrive at an outcome that describes meaningful types of users, we selected *a priori *four complementary survey indicators that measure multiple dimensions of potential SODIS-use (Table 1). In addition, we supplemented our SODIS-use indicators with two monitoring indicators (Table 1) to identify households that contributed limited information to the classification process due to infrequent observation. We used all six indicators to classify households into adoption groups (more below) to reduce the potential for reporting bias and misclassification error in SODIS-use behaviour.

**Table 1:** Indicators for SODIS-use

Indicator		Rational and Interpretation
***SODIS-use indicators***		

1.	*"Bottles sun-exposed"* Proportion of weeks during which SODIS bottles were observed to be exposed to sunlight (as observed by community-based staff)	Indicator for the intention to disinfect water using SODIS. Indirect indicator to measure actual use.

2.	*"Bottles ready-to-drink"* Proportion of weeks during which SODIS bottles were ready-to-drink (as observed by community-based staff)	Households regularly disinfecting water with SODIS usually have bottles of SODIS-treated water ready-to-drink available in-house. Considered to be a more reliable indicator for actual use than "bottles exposed to sunlight"

3.	*"Classified user"* Proportion of weeks during which a family was classified as SODIS-user (judgement of community-based staff after observing the family for at least 4 weeks).	Considered the most reliable indicator for actual use. Staff living in the community bases their judgement on daily observations of correct application, placing bottles in plain sunlight and/or getting drinking water from a SODIS-bottle when asked for.

4.	*"Behavioural change"* Regression coefficient of a logistic regression of the occurrence of bottles exposed to the sunlight (yes/no in a given week) *versus *time.	Indicates behavioural change over time. Coefficient reflects an increase (high values), decrease (low values) or constancy of exposing bottles to sun throughout monitoring time. Note: a coefficient of B = 0 indicates constant SODIS-use at high or low levels

***Monitoring indicators***		
5.	*"Time in Study - Bottles sun-exposed"* Total number of weeks during which *"Bottles exposed to sunlight" *was recorded	Discriminates and identifies households with few weeks observed.

6.	*"Time in Study - Classified user"* Total number of weeks during which *"Classified user" *was recorded	Discriminates and identifies households with few weeks observed to classify as SODIS-user.

### Statistical analysis

**Figure 1:**
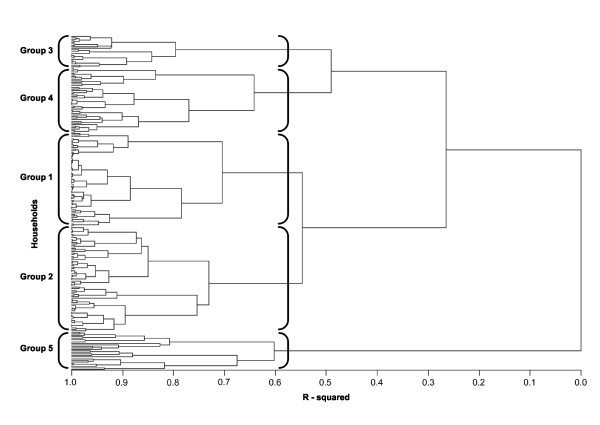
**Cluster analysis dendogram**. Horizontal axis denotes the linkage distance (R-square distance) between households according to their SODIS-use indicators listed in Table 1

To identify patterns of SODIS-use, we explored the multivariate distribution of study households in terms of the six quantitative SODIS-use indicators (Table 1) using principal component analysis [33]. Identification of meaningful SODIS-user groups was done by Ward's grouping algorithm using R-squared distances as the metric of similarity between households. The Ward's method proved to generate the best qualitative classification among several clustering algorithms tested. Five differentiated groups were identified by this approach (Figure 1). To confirm the patterns of SODIS-use we further examined the distribution of the study households in the data defined by the factorial axes of a principal component analysis based on the SODIS-use indicators [33].

SODIS implementation measures and community- and household level characteristics were tested for univariate differences between groups with the Fisher's exact test for binary data and the Kruskal-Wallis test for non-normally distributed quantitative data. Characteristics with (i) two-sided p-values smaller than 0.1, (ii) less than 25% of missing values (to avoid data sparseness problems), and (iii) no collinearity with other covariates were included in a multivariable, ordinal logistic model. The previously identified SODIS-user groups were used as the categorical-ordinal outcome variable ranging from "non-adopters" to "emerging-adopters". Robust standard errors were calculated to account for community level clustering. All analyses were performed in STATA 10 (StataCorp. 2007) and in SAS (SAS Institute Inc., Cary, NC, USA).

### Ethics

Ethical approval for this study was granted within the framework of the registered BoliviaWET cRCT (ClinicalTrials.gov Identifier: NCT00731497) [15]. The original trial whose data were used for this subgroup analysis was funded by the NIH and approved by the three humans subjects review boards involved, i.e. the Swiss ethics commission of the University of Basel, Switzerland, the University of California, Berkeley, and the University of San Simon, Cochabamba, Bolivia. Municipal authorities of Cochabamba and Totora also accepted the study and informed consent was obtained from community leaders, male and female household heads prior to implementation. All field staff completed training on research ethics (http://www.fhi.org/training/sp/Retc/). In addition, measures taken to meet ethical standards, including the processes to obtain necessary clearances and staff training, are described in the same publication [15].

## Results

### Intervention activities and compliance

The field-based monitoring staff assessed household intervention compliance weekly for a period of 42 weeks from June 2005 to June 2006 (median: 39 visits, IQR: 34-40).

At the community level, PCI conducted a total of 210 group events, which consisted of 108 community- (median 8/community, IQR: 7-12), 77 women- (median 7/community, IQR: 3-10), and 25 school-events (median 3/community, IQR: 1.5-3). During the study PCI conducted 2886 motivational household visits (median 12/household, IQR: 8-18).

The level of SODIS-use varied depending on the indicator used and the source of information. The community-based staff observed an overall median of 33% (IQR: 17-50) of households with SODIS bottles exposed to sunlight during weekly visits. The SODIS-implementing PCI staff registered during monthly household visits a median proportion of 75% (IQR: 60-85) of households with SODIS bottles exposed to the sun. After three months of intensive implementation, PCI staff recorded 77% of household respondents reporting regular SODIS-use, and 88% at the end of the study.

### SODIS-user group classification

Figure 1 summarizes the results of the cluster analysis, which identified five distinct SODIS-use groups based on household-level use indicators. Group 5 (25 households) differed from the other groups with respect to the time under observation (indicators 4 and 5): its time under observation (median 20 weeks, IQR: 16-23) was considered too short to obtain a valid estimate of SODIS-use and led to high variability in all of the indicators. Based on the limited information in group 5, we decided to exclude it from further analysis. Groups 3 and 4 comprised households with the highest SODIS-usage rates; group 3 with an initially high uptake and declining SODIS-use over time, group 4 with an emerging adoption pattern. Based on this group separation, we used characteristics of households in the groups to describe them in meaningful, qualitative terms: Group 1 = 'non-adopters', Group 2 = 'minimal-adopters', Group 3 = 'declining-adopters' and group 4 = 'emerging-adopters' (see Additional file 2).

**Figure 2:**
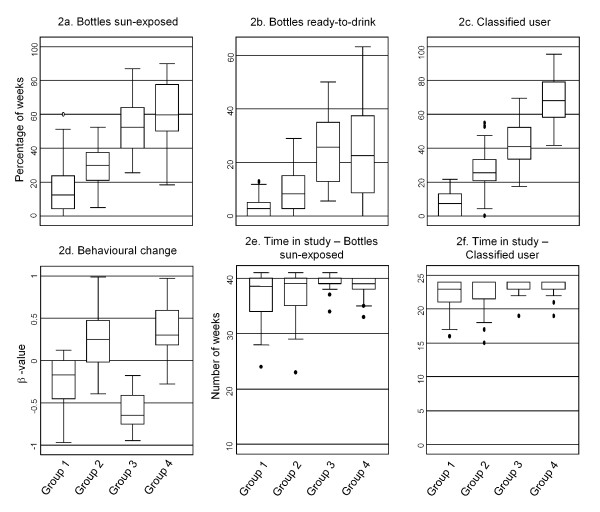
**Box-plots of four SODIS-user groups differing in six SODIS-use indicators (see Table **1)

**Figure 3:**
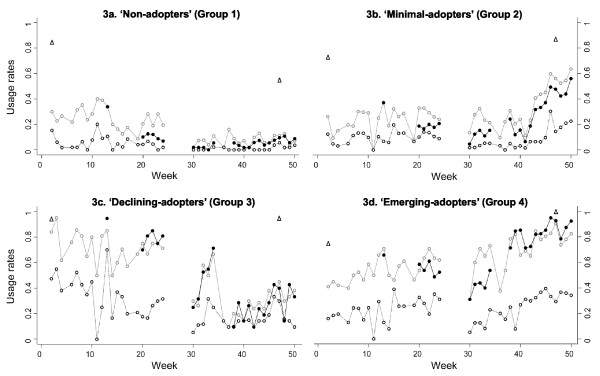
**Weekly observed proportion of households using SODIS in four SODIS-user groups**. Open triangles: self-reported SODIS-use at the beginning (after 3 month of initial SODIS promotion) and at the end of follow-up; filled dots: SODIS-use observed by project staff living in the community (see table 1 for definition); open grey circles: SODIS bottles observed on the roof; open black circles: SODIS bottles observed ready to drink

Figure 2 shows the difference between groups in four different SODIS-use indicators (self-reported and observed use) and two monitoring indicators (Table 1), and Figure 3 shows different SODIS-usage rates over time using the same indicators for the four user groups.

The group of 'non-adopters' consisted of households with little interest in adopting and using SODIS (median proportion of weeks with bottles exposed to sun were observed: 0.13; IQR: 0.04-0.24) (Figure 2a and 3a). 'Minimal-adopters' used SODIS more frequently: median proportion: 0.3 (IQR: 0.21-0.38) (Figure 2a and 3b) of the weeks observed. The 'declining- and emerging adopters' constituted the households with the highest SODIS-usage rates (median: 0.53 and 0.60; IQR: 0.40-0.64 and 0.50-0.78) (Figure 2a and 3c and 2d). 'Declining-adopters' used SODIS more often at the beginning of the follow-up (Indicator 4 "Behavioural change" in Table 1, logistic regression coefficient bottles exposed to sun vs. time) median: -0.65; IQR: -0.75-0.38 (Figure 2d and 3c). 'Emerging-adopters' used SODIS more often toward the end of the follow-up with a median of 0.30; IQR: 0.20-0.60 (Figure 2d and 3d).

### Factors influencing SODIS adoption

Table 2 includes the characteristics of the four different SODIS user groups. Some household characteristics differed significantly at a 95%-confidence level between SODIS-use groups. 'Emerging-adopters' consisted of more females compared to the other groups. 'Decreasing-adopters' were more likely to own bicycles. Households from both 'emerging-' and 'decreasing- adopter' groups were more likely to own a latrine (56% and 26%) than 'non- and minimal- adopters' households (both 8%). Further, they were more likely to have severely wasted children (two times substandard weight-for-height = 65% and 66%, respectively) than 'non-adopters' (17%) and 'minimal-adopters' (25%). Groups with the highest SODIS-usage rates lived in close proximity to their water source: the median distance was 5 m ('declining-adopters') and 10 m ('emerging-adopters'); in contrast, 'non-adopters' lived the furthest distance away from their water source with a median of 100 m, followed by the 'minimal-adopters' (30 m).

**Table 2:** Distribution of potential household determinants of SODIS-use

				**Groups based on SODIS-use behaviour**												
				
	**Total**			**Group 1** **('non-adopters')**			**Group 2** **('minimal-adopters')**			**Group 3** **('declining-adopters')**			**Group 4** **('emerging-adopters')**			
	**n = 216**			**n = 60**			**n = 68**			**n = 21**			**n = 42**			P- values*
	
**Demography**																
Number of household members	216	6.0	(5;9)	60	6.0	(5;8.5)	68	6.0	(4;9)	21	6.0	(5;7)	42	7.0	(5;9)	0.24
Age of household members	216	15.8	(13;18.1)	60	15.5	(13.7;17.6)	68	15.9	(13.3;18.7)	21	15.9	(13;17.8)	42	16.0	(12.1;18.4)	0.88
Number of females	216	3.0	(2;4)	60	3.0	(2;4)	68	3.0	(2;5)	21	3.0	(2;4)	42	4.0	(3;6)	**0.04**
Pregnant women at start of campaign	216	0.0	(0;0)	60	0.0	(0;0)	68	0.0	(0;0)	21	0.0	(0;0)	42	0.0	(0;0)	**0.09**
Children aged < 5	216	1.0	(1;2)	60	1.0	(1;2)	68	2.0	(1;2)	21	1.0	(1;2)	42	2.0	(1;2)	**0.06**
Children aged 5-9	216	1.0	(0;2)	60	2.0	(0;2)	68	1.0	(0;2)	21	1.0	(0;2)	42	1.0	(1;2)	0.60
Children aged 10-14	216	0.0	(0;1)	60	0.0	(0;2)	68	0.0	(0;1)	21	0.0	(0;1)	42	0.0	(0;2)	0.80
Members aged 15-19	216	0.0	(0;1)	60	0.0	(0;1)	68	1.0	(0;1)	21	0.0	(0;1)	42	0.0	(0;1)	0.95
Members aged > = 20	216	2.0	(2;2)	60	2.0	(2;2)	68	2.0	(2;2.5)	21	2.0	(2;2)	42	2.0	(2;2)	0.17
Caregivers' age	208	28.0	(23;36)	58	28.8	(23;35)	67	29.0	(23;37)	19	30.0	(22;36)	41	28.0	(23;40)	0.87
**Socioeconomic characteristics**																

Years of household heads' schooling	155	4.0	(3;5)	43	4.0	(2;5)	52	4.0	(3;5)	14	4.0	(3;5)	28	4.0	(2.5;5)	0.38
Monthly household income in US$	120	16.9	(0;37.5)	35	12.5	(0;25)	37	12.5	(0;31.3)	7	25.0	(12.5;37.5)	24	31.3	(0;47.5)	0.25
Bicycle: n (%)	192	107	(55.7)	49	25	(51)	65	40	(61.5)	18	14.0	(77.8)	39	17.0	(43.6)	**0.07**
Radio: n (%)	192	158	(82.3)	49	41	(83.7)	65	55	(84.6)	18	13	(72.2)	39	30	(76.9)	0.53
Gas cooker: n (%)	181	32	(17.7)	53	9	(17)	66	16	(24.2)	20	3	(15)	42	4	(9.5)	0.28
Number of rooms	192	3.0	(2;4)	49	2.0	(2;3)	65	3.0	(2;4)	18	3.0	(2;3)	39	3.0	(2;4)	0.29
Latrine: n (%)	192	34	(17.7)	49	4	(8.2)	65	5	(7.7)	18	10	(55.6)	39	10	(25.6)	**>0.001**
Electricity: n (%)	192	36	(18.8)	49	11	(22.5)	65	16	(24.6)	18	1	(5.6)	39	3	(7.7)	**0.06**
Solar panel: n (%)	130	30	(23)	29	9	(31)	50	8	(16)	12	3	(25)	25	5	(20)	0.44
Tiled roof: n (%)	181	57	(31.5)	53	19	(35.9)	66	18	(27.3)	20	8	(40)	42	12	(28.6)	0.60
**Environmental housing factors**																

Use of improved water source: n (%)**	192	149	(77.6)	49	36	(73.5)	65	53	(81.5)	18	15	(83.3)	39	29	(74.4)	0.69
Use of unimproved water source: n (%)***	192	133	(69.3)	49	37	(75.5)	65	48	(73.9)	18	10	(55.6)	39	24	(61.5)	0.23
Distance to water source in metres.	192	22.5	(5;50)	49	100.0	(10;200)	65	30.0	(7;100)	18	5.0	(4;30)	39	10.0	(5;200)	**0.03**
Turbidity of source water (NTU)	101	5.0	(5;20)	30	5.0	(5;40)	34	5.0	(5;20)	7	5.0	(5;40)	19	5.0	(5;15)	0.79
Faecal contamination of housing environment: n (%)	185	106	(57.3)	50	31	(62)	62	34	(54.8)	16	9	(56.3)	36	16	(44.4)	0.46
Animals present in the kitchen: n (%)	168	45	(26.8)	42	15	(35.7)	57	13	(22.8)	15	3	(20)	33	6	(18.2)	0.32
Soap, detergent present in the kitchen: n (%)	166	29	(17.5)	41	6	(14.6)	56	12	(21.4)	15	3	(20)	33	4	(12.1)	0.68
**Household members health status**																

Households with at least one stunted child < 5: n (%)	167	62	(37.1)	43	12	(27.9)	53	19	(35.9)	17	8	(47.1)	35	16	(45.7)	0.33
Households with at least one wasted child < 5: n (%)	167	85	(50.9)	43	17	(39.5)	53	25	(47.2)	17	11	(64.7)	35	23	(65.7)	**0.08**
Diarrhoea incidence in children < 5 before start of intervention	216	3.0	(0;7)	60	3.0	(0;5)	68	3.0	(1;7)	21	6.0	(1;12)	42	3.0	(0;6)	0.22
Diarrhoea prevalence (%) in children < 5 before start of intervention	216	7.0	(1;14)	60	5.0	(0;12)	68	8.0	(2;14)	21	7.0	(2;32)	42	6.0	(0;17)	0.26
Cough prevalence (%) in children < 5 before start of intervention	216	8.0	(0;20)	60	5.0	(0;17)	68	8.0	(2;21)	21	1.0	(0;17)	42	10.0	(0;24)	0.72
Fever prevalence (%) in children < 5 before start of intervention	216	7.0	(2;15)	60	6.0	(0;16)	68	5.0	(1;11)	21	6.0	(2;19)	42	7.0	(2;22)	0.58
**Hand-washing behaviour**																

Hand-washing per day of children > 5 and adults	169	4.0	(3;5)	44	4.0	(3;5)	57	3.0	(3;5)	15	3.0	(3;5)	33	4.0	(3;5)	0.27
Hand-washing per day of children < 5	192	2.6	(2;3)	49	2.5	(2;3)	65	2.5	(2;3)	18	3.0	(2;3)	39	2.7	(2;3)	0.96
**Household water management**																

Safe storage: n (%)	155	19	(12.3)	34	4	(11.8)	57	5	(8.8)	14	5	(37.5)	30	2	(6.7)	**0.06**
Water disinfection: n (%)	192	42	(21.9)	49	12	(4.5)	65	13	(20)	18	5	(27.8)	39	9	(23.1)	0.86
Household water consumption [l/household day]	189	40	(20;50)	58	35	(20;50)	67	40	(20;60)	21	50	(20;60)	41	30	(20;60)	0.81
Satisfied with quality of drinking water: n (%)	201	190	(94.5)	54	51	(94.4)	64	59	(92.2)	19	19	(94.7)	40	39	(97.5)	0.76

Baseline data are median (Q1; Q3), otherwise specified. *: Kruskal-Wallis and Fisher's exact test for comparing group 1, 2, 3, and 4; **: Improved water source: piped water into dwelling, plot or yard; tubewell/borehole; protected spring; rainwater collection. ***: Unimproved water source: unprotected dug well or spring; bowser-truck; surface water (river, dam, pond, irrigation channels)

Table 3 summarizes household exposure to the SODIS campaign through active participation at community-level events and through passive exposure to motivational activities during household visits. Since the implementation was standardised at community- and household levels there is no difference between the four SODIS-user groups regarding campaign features such as 'Number of events taken place per community', 'Average number of participants per event and community', and 'Number of household visits per household'. However, groups differed significantly regarding active participation at those events. 'Non-adopters' participated on average at half of the events offered, whereas 'declining and emerging adopters' participated at 78% and 71% of the events. The level of participation at school events was similar across groups, since participation was mandatory for school children in all schools in the study site.

**Table 3:** SODIS campaign at household and community level

				**Groups based on SODIS-use behaviour**												
				
	**Total**			**Group 1** **('non-adopters')**			**Group 2** **('minimal-adopters')**			**Group 3** **('declining-adopters')**			**Group 4** **('emerging-adopters')**			
	**n = 216**			**n = 60**			**n = 68**			**n = 21**			**n = 42**			P- values*
	
**Household exposure to SODIS campaign**																
Different events visited by at least one household member (n)	213	10.0	(6;13)	58	7.5	(6;12)	68	10.0	(6;12)	21	13.0	(9;17)	42	12.0	(7;14)	**0.002**
Events visited by at least one household member (n)	213	11.0	(6;15)	58	8.5	(6;14)	68	11.0	(6;15)	21	16.0	(11;22)	42	14.0	(10;18)	**0.004**
Proportion of possible events per community visited (%)	213	62.0	(39;83)	58	50.0	(32;80)	68	62.0	(44;81)	21	78.0	(57;100)	42	71.0	(48;94)	**0.017**
Events visited by most active household member (n)	213	6.0	(4;8)	58	5.0	(3;8)	68	5.5	(4;8)	21	9.0	(6;11)	42	6.0	(4;9)	**0.002**
Community events visited by at least one household member (n)	213	5.0	(3;7)	58	5.0	(3;7)	68	5.0	(3;9)	21	5.0	(3;6)	42	7.0	(5;9)	**0.019**
Women events visited by at least one household member (n)	213	2.0	(1;4)	58	2.0	(1;3)	68	2.0	(1;4)	21	7.0	(2;8)	42	3.0	(1;4)	**0.003**
School events visited by at least one household member (n)	213	0.0	(0;2)	58	0.0	(0;2)	68	0.0	(0;2)	21	0.0	(0;3)	42	0.0	(0;3)	0.515
Household visits by promoting NGO (n)	213	12.0	(8;18)	57	10.0	(6;19)	68	13.0	(9;18)	21	16.0	(12;21)	42	12.5	(9;18)	0.224
**SODIS campaign at community level**																

Events taken place per community (n)	216	18.0	(16;21)	60	19.0	(15.5;21)	68	18.0	(16.5;21)	21	21.0	(17;23)	42	17.5	(16;21)	**0.037**
Average number of participants per event per community	216	29.6	(23.2;40.4)	60	29.4	(20.1;40.4)	68	30.1	(24.0;48.8)	21	27.1	(27.1;30.1)	42	30.1	(27.1;40.4)	**0.071**
Average duration of events per community (hrs)	216	3.3	(2.9;3.8)	60	3.1	(2.8;3.6)	68	3.2	(2.8;3.7)	21	3.8	(3.4;3.8)	42	3.4	(3.1;3.8)	**0.018**

Data are median (Q1;Q3), otherwise specified. *: Kruskal-Wallis and Fisher's exact test for comparing group 1,2,3, and 4.

Since SODIS implementation indicators were correlated with each other, only one indicator ('Total number of events visited by at least one household member') was included in the multivariable model because it encapsulates the others. Estimates from the ordinal logistic model indicate that 'Total number of events visited by at least one household member' was positively associated with frequent SODIS use group membership (Table 4). For each additional event visited the odds of being in the next higher category of adoption was 1.07 (95% CI: 1.01-1.13). The multivariable model showed that higher adoption groups were more likely to own a latrine (OR: 3.38; 95% CI: 1.07-10.70) and to have at least one wasted child living in the household (OR: 2.17; 95% CI: 1.34-3.49). Furthermore, more females living in a household was positively associated with increased SODIS adoption (OR: 1.18; 95% CI: 1.07-1.30).

**Table 4:** Results of the multivariable ordinal logistic regression model

Predictor	Univariable model (n = 189) (SODIS implementation factor only)		
	OR	95% CI*	P value
			
Total no. of events visited by at least one household member	1.07	1.01-1.13	**0.02**
			

	**Multivariable model (n = 146)**		
	**OR**	95% CI*	**P value**

			
Total no. of events visited by at least one household member	1.04	0.98-1.11	0.15
Nr of females per household	1.18	1.07-1.30	**0.001**
Household with pregnant women at start of campaign	1.33	0.67-2.64	0.41
Bicycle ownership	0.75	0.35-1.64	0.48
Latrine	3.38	1.07-10.70	**0.04**
Distance (meters) to water source (log of)	0.94	0.73-1.22	0.65
Households with at least one wasted child under 5	2.17	1.34-3.49	**0.001**
			

* calculated from robust standard errors adjusted for community cluster

## Discussion

We characterised in a cluster analysis four distinct SODIS user groups after a 15-month comprehensive SODIS-dissemination campaign among the participants of a community-randomised, controlled SODIS-evaluation trial in rural Bolivia. Household characteristics that were most strongly associated with the adoption of the SODIS household water treatment method include the intensity of exposure to the SODIS campaign, the number of females per household, latrine ownership, and having severely wasted children living in the home. These three household characteristics that were strongly associated with SODIS-use may help to target SODIS promotion efforts to the population that would more easily adopt SODIS and would, thus, increase the impact of such efforts. The systematic identification of delivery strategies to improve compliance in HWT campaigns is important because improved compliance has consistently been associated with larger reductions in child diarrhoea across numerous HWT efficacy trials [2,3,5].

Our findings suggest that the motivation to adopt new water treatment habits and to acquire new knowledge about drinking water treatment is associated with prior health-related engagements, e.g. in latrine construction, and by with the experience of family health concerns such as living with an acutely malnourished child. In addition, higher SODIS-use was associated with the frequency of exposure to SODIS promotion of anyone of the household members. It is likely that eager adopters of new ideas and technological inventions such as SODIS are more interested in participating at the related promotional events.

Our findings are consistent with previous studies: In a similar setting in Bolivia, Moser and Mosler [25] found existing knowledge about the need to treat drinking water predicted early SODIS adoption. Applying the theory of the diffusion of innovations from Rogers et al. [34] in a SODIS diffusion programme in rural Bolivia they found that participation at SODIS-campaign events correlated positively with SODIS-use [24]. Further, a field study from Nicaragua reported that intention to use and actual use were related to a positive attitude toward the new technology [35]. These coherent findings on the motivating factors for SODIS adoption underscore the importance of determining a target population's characteristics and its attitude towards new technology prior to promoting SODIS.

The indicators we employed in our analysis to measure households' weekly SODIS-use were based on inconspicuous structured observations conducted by our community-based staff who were not involved in any SODIS-promotion activity. In combining objective indicators that measured visible signs of use (e.g. bottles exposed to sun) with proxies more responsive to the direction and magnitude of the change of treatment behaviour (e.g. weekly observation of correct application of SODIS), we increased the quality of measurement and reduced the potential for reporting bias and misclassification error [26-28]. Our independent evaluation of SODIS-use generated much lower adoption rates than estimates from the implementing organization, PCI (32% versus 75%). This underscores the potential for bias in situations when implementers evaluate their own work. Such courtesy bias and over-reporting of compliance with the intervention is well known from water, sanitation and hygiene intervention studies [7,26,36-42]. The discrepancy between the levels of SODIS compliance assessed through different indicators in our study raises questions about the consistency of compliance rates reported in prior studies in peer-reviewed and grey literature. Our results highlight the importance of choosing independent staff and a valid and responsive indicator to assess use and to draw conclusions about the implementation effectiveness of HWT intervention programmes.

Despite an intensive 15-month promotion campaign carried out by a highly qualified implementing organization, we observed 32% overall compliance with the solar water disinfection method during our 12 months of follow-up [15]. Our findings suggest that SODIS promotion would benefit from re-assessing the core marketing messages and approaches to reach the critical 50% fraction of early and willing SODIS adopters in the population [25]. Our analysis identified some characteristics associated with frequent use. However, it is the characteristics of willing but occasional user groups (our 'minimal adopters') to whom new marketing and promotion strategies should be targeted [43]. Based on the characteristics that we measured, it was difficult to differentiate the 'minimal adopters' from 'non-adopters' (Table 2). In this population, the 'non-adopter' and 'minimal-adopter' groups included the most marginalized households by observable characteristics: they were poorer, lived further from water sources, rarely owned a latrine, had more frequently faecally contaminated home environments, and had more animals roaming their kitchen area; yet, unexpectedly, they were less likely to have wasted children in their families (Table 2).

Criteria to plan for the successful roll-out and targeting of water and sanitation programmes based on demand-responsive approaches have often been suggested [44]. In the Bolivian context, SODIS-programme planning may benefit from assessing easy measurable household-level factors like the latrine ownership, a large proportion of females and the presence of a malnourished child to identify population subgroups that can be targeted for rapid uptake of the SODIS HWT method. Those insights supported by our data are consistent with recommendations for a successful roll-out of water and sanitation programmes deriving from previous studies [45-47].

There are limitations to this study. The participating communities were not homogenous regarding pre-existing water supplies and sanitation infrastructures, previous exposure to sanitation and hygiene campaigns, as well as political support to participate in the study. Further, the ordinal logistic regression assumes that the categories follow an intrinsic order. This order is evident for 'non- and minimal adopters' but is less obvious in the case of 'declining- and emerging-adopters'. We felt the ordinal grouping was justified because from the programme-implementation viewpoint the sustained users (the 'emerging adopters' in this analysis) are the most valuable group for sustained impact [34]. To ensure that our findings were not sensitive to the modelling approach, we repeated the analysis using multinomial regression, which does not impose an order to the categorical outcome. Analogous to our presented results, the multinomial regression identified latrine ownership and presence of severely wasted children as the most important predictors of SODIS-use categories (results available from the authors). Finally, data on the SODIS-use indicator 'Households rated as SODIS-user by implementation-independent field worker', was incomplete because (i) the indicator was implemented after an intensive 3-month pilot phase, and (ii) it required the randomly-rotated field staff (every three months) to familiarize themselves with each local community for a period of four weeks before they could report the indicator [15]. While we believe this measure reduced systematic reporting bias and enhanced the reliability of SODIS-use measurement, it reduced the total observation time available for analysis.

## Conclusions

Analyses of implementation effectiveness and the dynamics of SODIS-uptake from large- scale SODIS dissemination programmes are rarely published. Our findings suggest that households that have more women, own a latrine, have malnourished (wasted) children and are close to their water source are more likely to adopt SODIS during an intensive promotion campaign. Households that did not adopt SODIS tended to be poorer, further from water sources and having less hygienic home environments. This finding suggests how implementers could identify populations most likely initially to begin SODIS use and to sustain its use over time.

## Competing interests

The authors declare that they have no competing interests.

## Authors' contributions

AC and DM conceived the idea and developed the design for the study. AC wrote the original draft manuscript, and incorporated revisions from each of the co-authors. GDP and JH contributed to the conception and design of the manuscript and conducted the statistical analysis. AC and MC coordinated and supervised data acquisition. DM, JH, GDP, and BFA wrote parts of the paper and together with, MC, JMC, and SI contributed to the conception of the manuscript and provided revisions. All authors read and approved the final manuscript.

## Pre-publication history

The pre-publication history for this paper can be accessed here:

http://www.biomedcentral.com/1471-2458/11/210/prepub

## Supplementary Material

Additional File 1 **SODIS promotion and implementation scheme (based on Perera *et al. *2007)**. Source: Mäusezahl D, et al. (2009) Solar Drinking Water Disinfection (SODIS) to Reduce Childhood Diarrhoea in Rural Bolivia: A Cluster-Randomized, Controlled Trial. PLoS Med 6(8): e1000125. Click here for file

Additional File 2 **3D scatter plot view of SODIS user groups of the first three principal components**. Click here for file
