# Supplementary figures and images for: Factors associated with compliance among users of solar water disinfection in rural Bolivia
Source: BMC Public Health. 2011 Apr 4;11:210. doi: 10.1186/1471-2458-11-210 (PMC3098791; doi:10.1186/1471-2458-11-210)

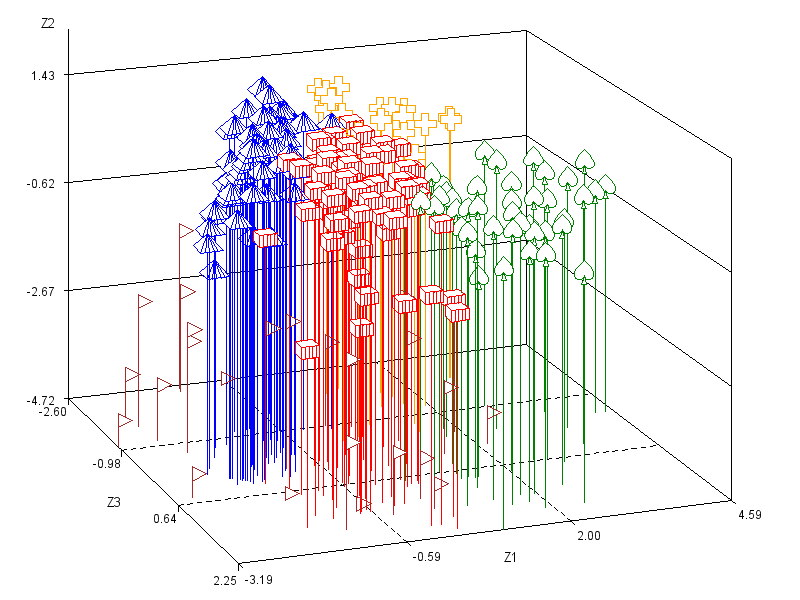

Supplement: Additional File 2 — 3D scatter plot view of SODIS user groups of the first three principal components. [file 1471-2458-11-210-S2.GIF]
